# Single-cell sequencing analysis reveals cancer-associated pericyte subgroup in esophageal squamous cell carcinoma to predict prognosis

**DOI:** 10.3389/fimmu.2024.1474673

**Published:** 2025-01-06

**Authors:** Kai Xiong, Bing Pan, Hao Fang, Ziyou Tao

**Affiliations:** ^1^ Department of Cardiothoracic Surgery, Tianjin Medical University General Hospital, Tianjin, China; ^2^ Tianjia Genomes Tech Cor. Ltd., Hefei, China; ^3^ Department of Thoracic Surgery, the First Affiliated Hospital of Zhengzhou University, Zhengzhou, China

**Keywords:** single-cell sequencing, prognosis, esophageal squamous cell carcinoma, pericyte, cell communication

## Abstract

**Background:**

The role of cancer-associated pericytes (CAPs) in tumor microenvironment (TME) suggests that they are potential targets for cancer treatment. The mechanism of CAP heterogeneity in esophageal squamous cell carcinoma (ESCC) remains unclear, which has limited the development of treatments for tumors through CAPs. Therefore, a comprehensive understanding of the classification, function, cellular communication and spatial distribution of CAP subpopulations in ESCC is urgently needed.

**Methods:**

This study used large-sample single-cell transcriptome sequencing (scRNA-seq) data to investigate pericytes’ subpopulation characteristics, functions, upstream and downstream regulation and interactions with other components of the TME in the ESCC, and analyzed prognostically in conjunction with Bulk RNA-seq data. In addition, pericyte subpopulations were validated and their spatial distribution in the ESCC TME was observed by multiplex immunofluorescence. Drug prediction and molecular docking was further used to validate the medicinal value of drug targets.

**Results:**

CAPs in the ESCC TME were found to be highly heterogeneous, and we identified six pericyte subtypes: c1_ARHGDIB, c2_BCAM, c3_LUM, c4_SOD2, c5_TYMS, and c6_KRT17, which have commonality in a part of their functions, and each of them has a major function to play, by having different strengths of interaction with different components in the TME. In addition, we found that c4_SOD2 was negatively correlated with prognosis, conversely, c5_TYMS was positively correlated with prognosis. The drug with a better effect on c5_TYMS was docetaxel (binding energy = -8.1, -8.7 kcal/mol); raloxifene may be more effective against c4_SOD2, although raloxifene has a slightly lower binding energy to SOD2 (-6.4 kcal/mol), it has a higher binding energy to PDGFRβ (-8.1 kcal/mol).

**Conclusion:**

The present study identified and discovered pericyte subpopulations that were significantly associated with prognosis, which provides new biomarkers for predicting patient prognosis and adds usable targets for immunotherapy, and it is also important for gaining insights into the composition of the TME in ESCC.

## 1 Introduction

In recent years, progress has been made in the diagnosis and treatment of esophageal squamous cell carcinoma (ESCC) (1). However, a significant proportion of patients do not respond well to immunotherapy, which largely stems from the fact that researchers do not yet have a deep enough and comprehensive understanding of the complex tumor microenvironment (TME) of ESCC, in particular, little research has been carried out on the pericytes which are closely related to tumor angiogenesis (2, 3).

Pericytes are a type of mural cell located between the capillary endothelium and the basement membrane, and they play a role in regulating capillary vasomotion and maintaining normal microcirculation in local tissues and organs. In the tumor microenvironment, pericytes have multiple interactions with different components, such as constituting pre-metastatic ecological niches, promoting cancer cell growth and drug resistance through paracrine activities, and inducing M2-type macrophage polarization (4). Some investigators have identified multiple non-immune mesenchymal cell subtypes including pericytes by using single-cell transcriptome sequencing, and the interactions between these cells and tumor cells and other cell types, and their role in the TME. interactions, as well as their immunosuppressive status in TME, further tell us that the role played by pericytes in the development and treatment of ESCC cannot be ignored (5).

Therefore, exploring the subpopulations of pericytes, their functions and the spatial distribution of pericytes in the TME are crucial for optimizing immunotherapy.

**Figure 1:**
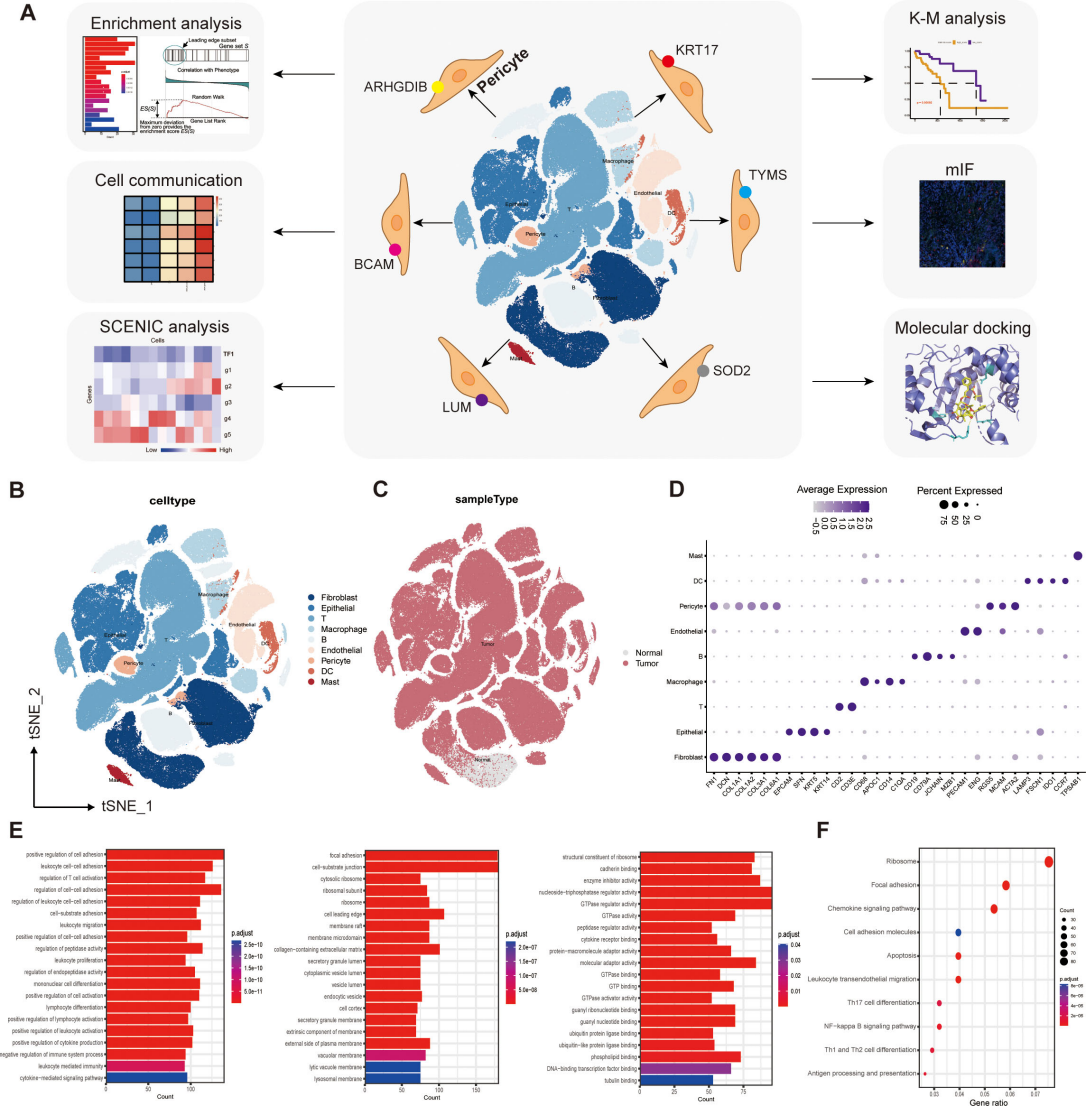
Single-cell transcriptome mapping and functional enrichment analysis. **(A)** Flow chart. **(B)** We captured the transcriptomes of 9 major cell types according to the expression of canonical gene markers. **(C)** T-SNE plots comparing the distribution of single cells derived from tumor and normal samples. **(D)** Annotated correspondence between markers and cellular subgroups. **(E)** GO enrichment results for differential genes. **(F)** KEGG enrichment results for differential genes.

Based on a large amount of single-cell sequencing (scRNA-seq) data, we identified for the first time that there are six pericyte subtypes in the TME of ESCC, and they differ in function and spatial distribution. We sorted out these differentiated subpopulations by a cluster-extract-recluster approach, and predicted the functions each of them exerted by detailed comparison and analysis of the functional enrichment results. To explore the pericyte-associated mechanisms more comprehensively, we also identified the transcription factors and target genes of these subtypes by SCENIC analysis, and analyzed the interactions of the different subtypes with other cell populations in the TME by cellular communication. We also analyzed a protective pericyte subpopulation and a cancer-promoting pericyte subpopulation related to concerning prognosis. Finally, we validated and spatially distributed these pericyte subtypes using multiplex immunofluorescence (mIF) in tumor tissue samples from clinical patients. In addition, we have predicted drugs with potential roles in regulating pericyte subpopulations and have used molecular docking to predict binding modes and molecular binding energies of proteins encoded by target genes to small molecule drugs. Our main research efforts and processes are presented in **Figure 1A**.

## 2 Methods

### 2.1 Sequencing data acquisition and analysis

We obtained scRNA-seq data for 64 purified ESCC tumor samples in GSE160269 dataset from the Gene Expression Omnibus (GEO) database (http://www.ncbi.nlm.nih.gov/geo/). Expression matrices for CD45+ and CD45- were obtained, and 64 CD45+ and 64 CD45- samples were combined into 64 samples. Bulk-RNA sequencing data and clinical information for ESCC patients were obtained from The Cancer Genome Atlas (TCGA) database (https://portal.gdc.cancer.gov), including 95 patients. We used data from the TCGA database as external validation sets. For patients with a survival outcome, the time from treatment to the most recent follow-up was defined as the survival time; for patients with a mortality outcome, the time from treatment to death was defined as the survival time.

The “Seurat” package in R (6) converted scRNA-seq data into Seurat objects. First, we checked the scRNA-seq data for quality control by deleting clusters with less than 3 cells, cells with less than 50 gene mappings and cells with over 5% of mitochondrial genes. Then, the “NormalizeData” package in R was applied for data normalization. With the first 2000 high-resolution genes, 26 principal components (PCs) were selected by principal component analysis (PCA). T-distributed stochastic neighbor embedding (t-SNE) was utilized for unsupervised clustering and unbiased visualization of cell subpopulations on a two-dimensional map (7). Clustree allows for visualization of clustering structures at multiple resolutions, helping in the selection of a parameter that maintains biologically meaningful cell type distinctions and clustering stability. By examining the Clustree plots, we selected a resolution of 0.2, as it provided a balance between the number of clusters and biological interpretability, avoiding over- or under-clustering while ensuring stability in downstream analyses. The “FindAllMarkers” function was utilized to compare the differences in gene-expression between one cluster and all other clusters to be sure the marker genes for each cluster (|log2FC| > 1, adj.p-value< 0.05). Subsequently, the most central step, we manually annotated the cellular subgroups by comparing the expression levels of the cellular markers.

### 2.2 CNV inference and cluster analysis of pericytes based on scRNA-seq data

The copykat R package (8) was used to identify diploid and aneuploid epithelial cells with default parameters.

We extracted the cells contained in the pericyte subpopulation from all the single cells obtained according to the previous cell annotation results, with the resolution set to 0.2, and used the same method to perform the cluster analysis, and then performed the difference analysis on the new subpopulation derived from the clustering, taking the top10 difference genes to draw the dot plot, and searching for the expressed characteristic genes to annotate the new subpopulation respectively.

We used the ‘CellCycleScoring’ function in Seurat to identify cell cycle phase-specific changes in different cell clusters. The ‘CellCycleScoring’ function assigns each cell a score based on the expression of G2/M and S phase markers. The G2/M or S phase scores were inversely correlated, and cells that did not express G2/M and S phase markers were in the G1 phase. The ‘CellCycleScoring’ function will assign each cell a predicted classification based on its score.

### 2.3 Functional enrichment analysis

We used Gene Ontology (GO) and Kyoto Encyclopedia of Genes and Genomes (KEGG) to enrich the differential genes of these pericellular subtypes to predict their functional differences (9), and in addition, we used GSEA on the gene expression matrices of each of these subtypes to assess whether there was a statistically significant difference in the expression patterns of the predefined genomes between the two predefined biological states (10, 11). We synthesized these enrichment results together to make functional predictions.

### 2.4 Cell communication analysis of pericyte subpopulations and SCENIC analysis

Cell communication analysis was performed on the top 10 tumor samples separately using cellphoneDB (12) software, and the threshold of significant intercellular interactions was p<0.01.

The SCENIC analysis was run as described (13), using the pyscenic (version 0.9.19) and hg19-500bp-upstream-10species databases for RcisTarget, GRNboost, and AUCell. The input matrix was the normalized expression matrix from Seurat.

### 2.5 Immunofluorescence staining of tissue sections

Multiple fluorescence immunohistochemistry kit (Panovue, Beijing, China), including containment reagents, TSA signal amplification solution, dyes, etc. Incubate overnight at 4°C with purified rabbit anti-human PDGFRβ (ab32570, 1:100, Abcam, USA), rabbit anti-human SOD2 (66474-1-Ig, 1:300, Proteintech, Wuhan, China), rabbit anti-human CD68 (66231-2-Ig, 1:2000, Proteintech, Wuhan, China), rabbit anti-human TYMS (66725-1-Ig, 1:200, Proteintech, Wuhan, China), mouse anti-human α-SMA(67735-1-Ig, 1:400, Proteintech, Wuhan, China) and purified rabbit anti-human EPCAM (21050-1-AP, 1:1000, Proteintech, Wuhan, China). Tissue sections were placed in an oven for 30 minutes, followed by two 10-minute immersions in xylene for dewaxing. After completion of dewaxing by a series of ethanol washes at decreasing concentrations, the sections were immersed in sodium citrate buffer for 15 minutes of microwave treatment for antigen repair, followed by natural cooling to room temperature. After completion of cooling, the sections were blocked by the addition of a blocking solution for 10 minutes, and the primary antibody was added and left for 4 overnight. Then add the appropriate amount of goat anti-mouse or rabbit secondary antibody to bind the primary antibody for 1 hour at 37°C. Corresponding dyes were then added for staining to move on to the next round of antibody staining. The intermediate bridging step was 1× TBST rinsing 3 times for 5 minutes each. DAPI was used to stain the cell nuclei. Fluorescence was detected by confocal microscopy (Olympus Company, Japan).

### 2.6 Drug prediction and molecular docking

Evaluating protein-drug interactions is important to understand whether a target gene can be used as an actual drug target. In this study, we will utilize the Drug Signature Database (DSigDB, https://dsigdb.tanlab.org/DSigDBv1.0/) in the Enrichr database (https://maayanlab.cloud/Enrichr/enrich/) to predict small molecule drugs that may act on drug target genes (14). And in principle, our screening conditions are adjusted P-value < 0.05.

Molecular docking simulations allow us to analyze the binding affinity and interaction patterns between ligands and drug targets. By identifying ligands with high binding affinity and favorable interaction patterns, it facilitates the next step of experimental validation and optimizes the design of potential drug candidates. In this study, Autodock Vina 1.1.2 (http://autodock.scripps.edu/), a computerized protein-ligand docking software for molecular docking of small molecule drugs and proteins encoded by the corresponding target genes, was used (15). Drug structure data were obtained from PubChem Compound Database (https://pubchem.ncbi.nlm.nih.gov/), and protein structure data were downloaded from PDB (Protein Data Bank, https://www.rcsb.org/). First, water molecules are first removed from the protein and ligand, then polar hydrogen atoms are added, and subsequently, the protein is wrapped in a grid box. After the docking was completed, the best bound conformation was selected for presentation in Pymol 3.0 (https://pymol.org/).

### 2.7 Statistical analysis

Statistical analyses were computed using R software 4.2.1 runs, differential gene analysis, correlation analysis, and survival curves were performed based on the “edgeR” package, the “psych” package, and the Kaplan-Meier “survival” package, respectively. GSEA software 4.3.2 runtime version (www.gsea-msigdb.org/gsea) was used for GSEA enrichment analysis. p < 0.05 was considered statistically significant. Statistical software Image J and Graphad Prism 9.5 were used to analyze the data. For variables obeying a normal distribution, we quantified differences by two-tailed t-tests or one-way ANOVA, as appropriate. If the data presented a non-normal distribution, we evaluated the differences using the Wilcoxon test or the Kruskal-Wallis test. It is important to emphasize that all statistical analyses were performed in an R programming environment with strict adherence to a p-value threshold of less than 0.05.

## 3 Results

### 3.1 Identification of pericyte-associated differential genes and enrichment analysis based on single-cell sequencing data

In total, we obtained 202,228 single cells from the sample. T-SNE was computed using 20 dimensions (1–20), and 26 clusters were further obtained using the ‘‘FindClusters’’ function with a resolution setting of 0.6, the main cell lineages were annotated by canonical lineage markers. The clustered subpopulations were manually annotated with markers for cell types as follows:

Epithelial cell: “EPCAM”,”SFN”, “KRT5”,”KRT14”;

Fibroblast: “FN1”, “DCN”, “COL1A1”, “COL1A2”, “COL3A1”,”COL6A1”;

Endothelial cell: “VWF”, “PECAM1”, “ENG”, “CDH5”;

Pericyte: “RGS5”, “MCAM”, “ACTA2”;

T cell: “CD2”, “CD3D”, “CD3E”, “CD3G”;

B cell: “CD19”, “CD79A”, “MS4A1”, “JCHAIN”, “MZB1”;

DC cell: “LAMP3”, “FSCN1”, “IDO1”, “CCR7”;

Mast cell: “TPSAB1”;

Myeloid cell: “CD68”, “LYZ”, “CD14”, “IL3RA”, “LAMP3”, “CLEC4C”, “TPSAB1”.

We captured the transcriptomes of 7 major cell types according to the expression of canonical gene markers. These cells included T cells, B cells, myeloid cells, endothelial cells (ECs), fibroblasts, epithelial cells, and pericytes (**Figure 1B**). T-SNE plots compare the distribution of single cells derived from tumor and normal samples (**Figure 1C**). The annotated correspondence between the most typical markers and cell subtypes is presented in the dot plot (**Figure 1D**).

Comparison of gene expression differences between pericyte clusters and all other clusters using the “FindAllMarkers” function identified 936 genes specifically expressed by pericyte subpopulations (|log2FC|≥0.25, min.pct≥0.1, test.use= “Wilcox”, FDR ≤ 0.05),. These genes were then subjected to GO enrichment analysis and KEGG enrichment analysis. The results of GO enrichment showed cell adhesion, regulation of lymphocyte differentiation and activation, regulation of immune system processes, activity of multiple organelles and their outer membranes, and multiple enzyme regulators (**Figure 1E**).

KEGG enrichment analysis showed that pericyte marker genes were mainly involved in the NF-κB signaling pathway, antigen processing and presentation, differentiation of Th1, Th2, and Th17 cells, cell migration, adhesion, and apoptosis (**Figure 1F**).

### 3.2 Identifying six pericyte subpopulations, two of which were significantly associated with prognosis

**Figure 2:**
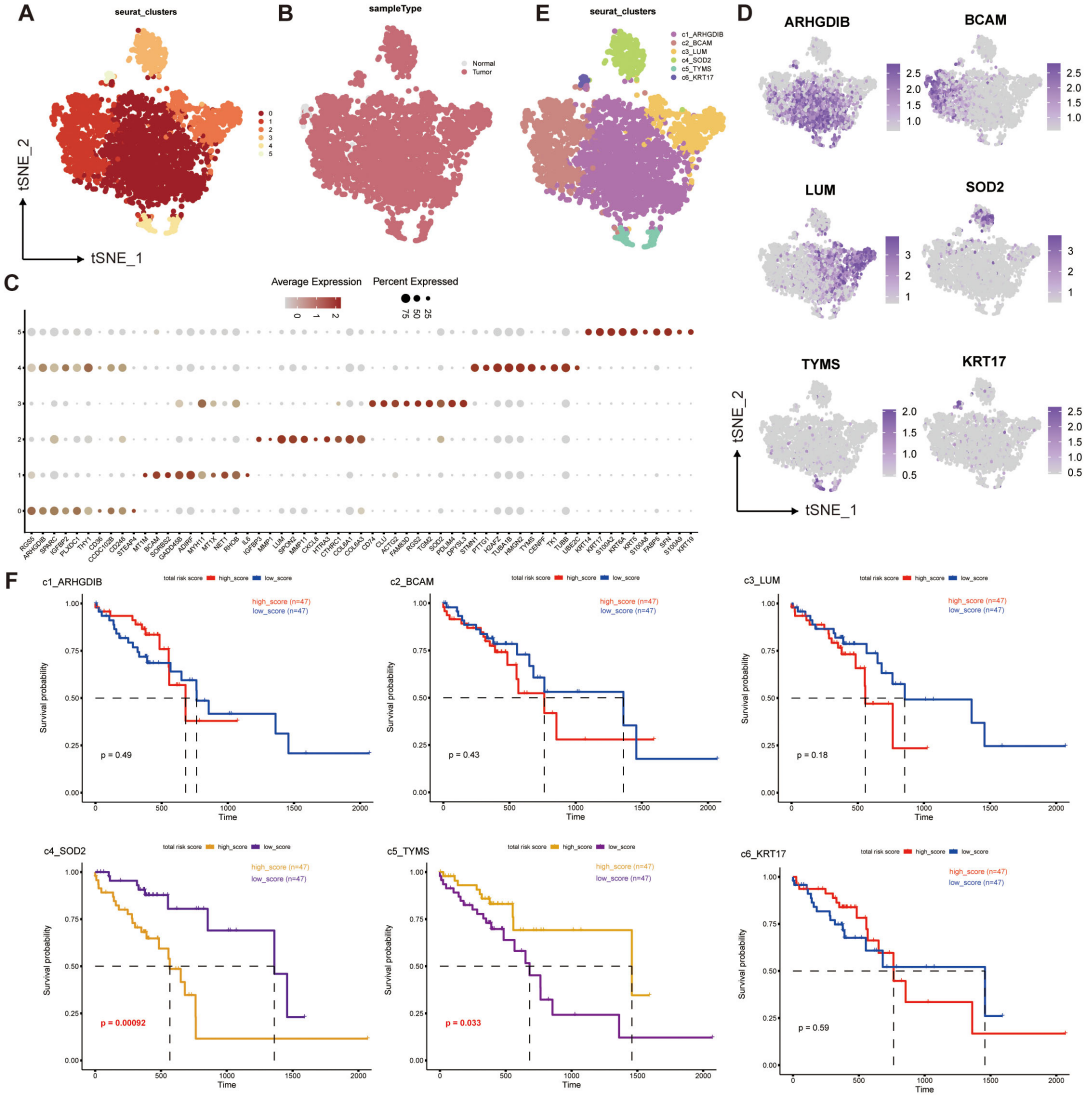
Single-cell transcriptome mapping and CNV analysis of ESCC multicellular ecosystems. **(A–E)** Distribution of pericytes in tumor and normal tissue samples, and clustering and annotation of their subpopulations. **(F)** Survival analysis of the corresponding scores of six pericyte subpopulations and prognosis of ESCC patients.

We extracted 4044 pericytes from 202,228 cells, and clustered them into 6 pericyte subpopulations (**Figure 2A**). We found that only 77 of these cells in the entire pericyte subpopulation were from normal samples (**Figure 2B**), suggesting that most of our pericytes may be in the tumor tissue or even in the tumor microenvironment, which may be relevant to prognosis. Subsequently, we performed differential gene analysis for the six subgroups, took the top 10 differential genes to plot a dot plot (**Figure 2C**), and identified the characteristic genes of the six subgroups: ARHGDIB, BCAM, LUM, SOD2, TYMS, KRT17, and annotated these genes according to the locations of the subgroups corresponding to their high expression in the T-SNE plot (**Figure 2D**). In this way, we obtained six pericyte subpopulations: c1_ARHGDIB, c2_BCAM, c3_LUM, c4_SOD2, c5_TYMS, c6_KRT17 (**Figure 2E**).

We performed a survival analysis of the genes characterizing the top50 of the six subpopulations of pericytes combined with the prognostic information of the patients (**Figure 2F**), and found that c4_SOD2 and c5_TYMS were significantly different from prognosis. Interestingly, c4_SOD2 was negatively correlated with prognosis, while c5_TYMS was positively correlated with prognosis. This suggests that c5_TYMS may be a protective cell subpopulation in the ESCC tumor microenvironment, whereas c4_SOD2 may be a cell subpopulation that contributes to the hostile environment, which is largely consistent with the results of our corresponding functional enrichment analyses, corroborating the scientific validity of our subpopulation annotations. Therefore, we preliminarily defined c4_SOD2 as suppressive cancer-associated pericytes (sup-CAPs) and c5_TYMS as promoted cancer-associated pericytes (pro-CAPs).

### 3.3 Mining the functions and downstream pathways of pericyte subpopulations

**Figure 3:**
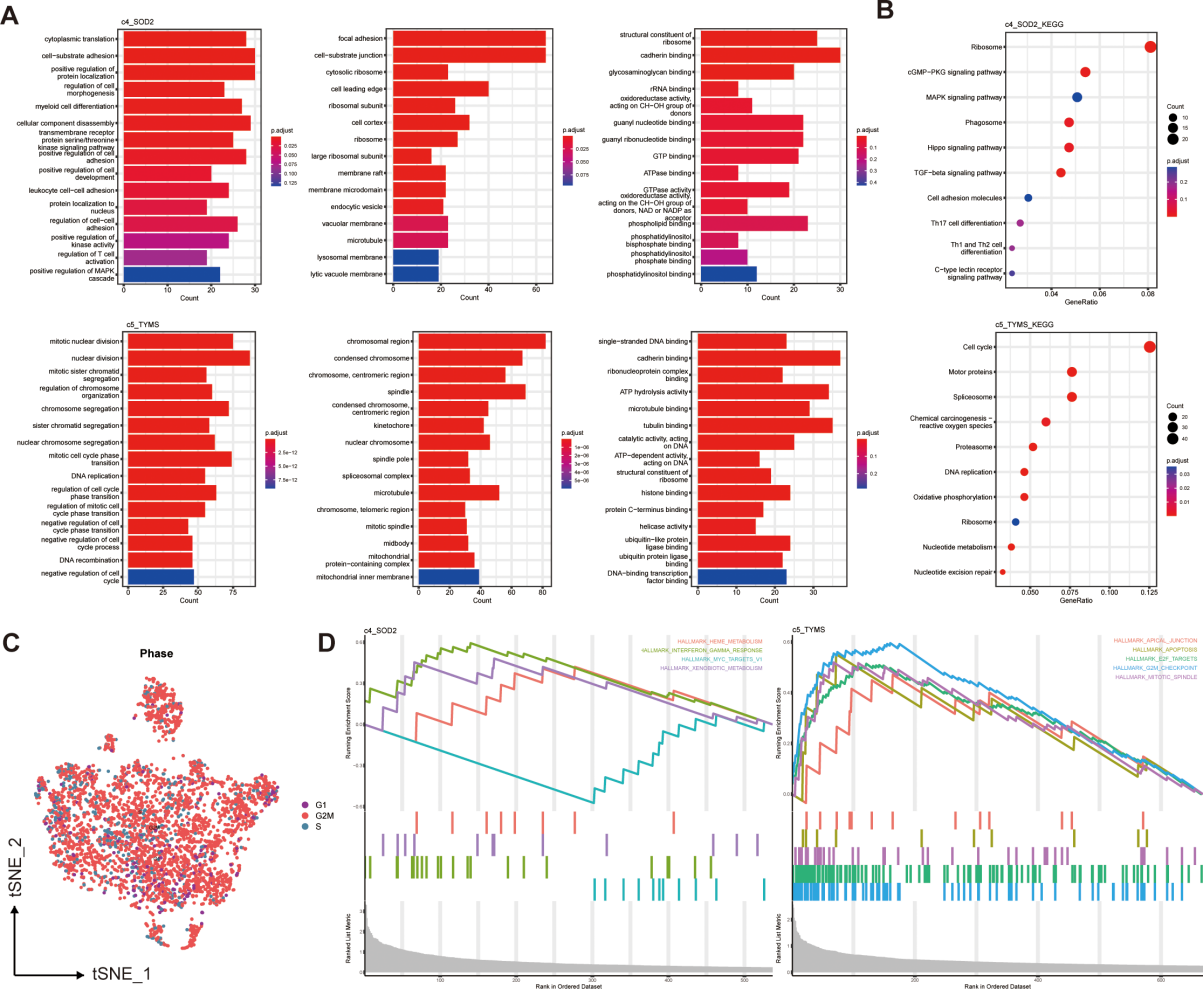
Functional enrichment of two prognostically relevant pericyte subpopulations. **(A)** GO enrichment results. **(B)** KEGG enrichment results. **(C)** Cell cycle distribution of all pericytes. **(D)** GSEA enrichment results.

To predict the functions of these six pericyte subpopulations as well as to mine their downstream pathways, we did pathway enrichment analysis on the characterized genes of the six subpopulations. The GO bar plots showed the top 15 enriched results in each of the three categories of biological processes (BP), cellular components (CC), and molecular functions (MF), c4_SOD2 was mainly enriched in cell adhesion, regulation of cell development, differentiation and activation, ATPase, GTPase and binding of multiple compounds; c5_TYMS was mainly enriched in mitosis or some mitochondria-related pathways, DNA replication, recombination, binding, and negative regulation of the cell cycle (**Figure 3A**).

Based on the KEGG enrichment analysis and KEGG bubble map showed the top 10 pathways, including Cell cycle(c5_TYMS), Th17,Th1 and Th2 cell differentiation (c4_SOD2) (**Figure 3B**). The results of GO and KEGG enrichment analyses of four additional subpopulations are displayed in **Supplementary Figures S1A, B**. The different pathways of each pericyte subpopulation were analyzed using GSEA. In the hallmark gene set, the GSEA enrichment plot (**Figure 3D**) showed the first 4 active pathways in c4_SOD2 and the first 5 active pathways in c5_TYMS, indicating that this pathway, TNF-γ response, was significantly enriched in c4_SOD2, whereas the pathways G2/M checkpoint, E2F target, mitotic spindle, and apoptosis pathways were significantly enriched in c5_TYMS, and the enrichment results of c1_ARHGDIB and c3_LUM are shown in **Supplementary Figure S1C**. Unfortunately, c2_BCAM and c6_KRT17 were not enriched for statistically significant pathways. We also performed enrichment analysis on the immune signature gene set and listed the active pathways with the top 5 normalized enrichment scores respectively (**Supplementary Figure S1D**), which will not be detailed here. The results of the functional enrichment analysis can reveal the underlying mechanisms or key loci of disease onset and progression, which can help us to study the therapeutic approaches in greater depth and improve the prognosis of clinical patients. We also observed the cell cycle of these pericytes, most of which were in the G2/M phase (**Figure 3C**). Because we concluded from the subsequent survival analysis that two subpopulations, c4_SOD2 and c5_TYMS, were significantly correlated with patient prognosis, only the enrichment results of these two subpopulations are shown and described here, and the rest will be discussed later.

### 3.4 Probing cellular communication and upstream-downstream analysis of pericyte subpopulations in the tumor microenvironment

**Figure 4:**
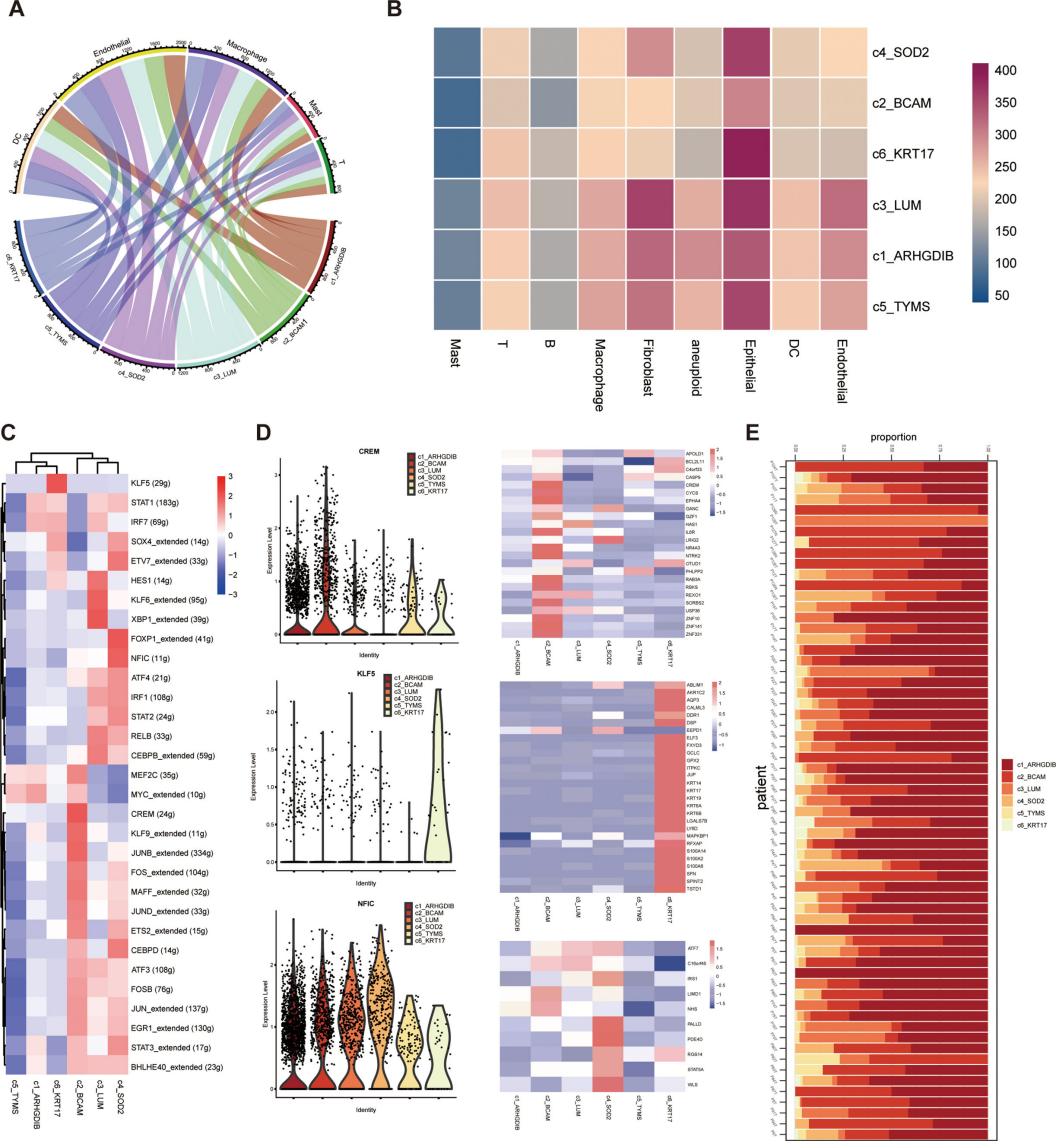
Probing cellular communication and upstream-downstream analysis of pericyte subpopulations in the tumor microenvironment. **(A, B)** Six pericyte subpopulations and cellular communication of different immune cells. **(C, D)** Results of SCENIC analysis of pericyte subpopulations. **(E)** Distribution of six pericyte subpopulations across all samples of single-cell sequencing data.

The results showed that these six pericyte subpopulations differed somewhat in their cellular communication with endothelial cells, myeloid cells, and other cell types in the tumor microenvironment. All pericyte subpopulations had very weak communication with T cells and giant cells, which indicated that pericytes did not have a close functional relationship with these two cell types; yet they all had strong communication with endothelial cells, especially c3_LUM and c4_SOD2 had the strongest communication with endothelial cells (**Figures 4A, B**). To dig deeper into the information about interactions between cell types, we also investigated which interaction-specific proteins exist between these pericyte subpopulations and other cell types, and in which cell types such interactions are significantly enriched. We further applied the single-cell regulatory network inference and clustering (SCENIC) approach to explore the transcription factors and their target genes that may regulate the development and differentiation of these six pericyte subtypes (**Figures 4C, D**). Transcription factors significantly highly expressed in these six pericyte subpopulations were MYC_extended (c1_ARHGDIB and c5_TYMS), CREM (c2_BCAM), XBP1_extended (c3_LUM), NFIC (c4_SOD2), KLF5 (c6_KRT17).

We also presented the proportions of each of the 6 pericyte subpopulations relative to all pericytes in each of the 64 tumor tissue samples (**Figure 4E**).

### 3.5 Multiple immunofluorescence stain of tissue samples from ESCC patients

**Figure 5:**
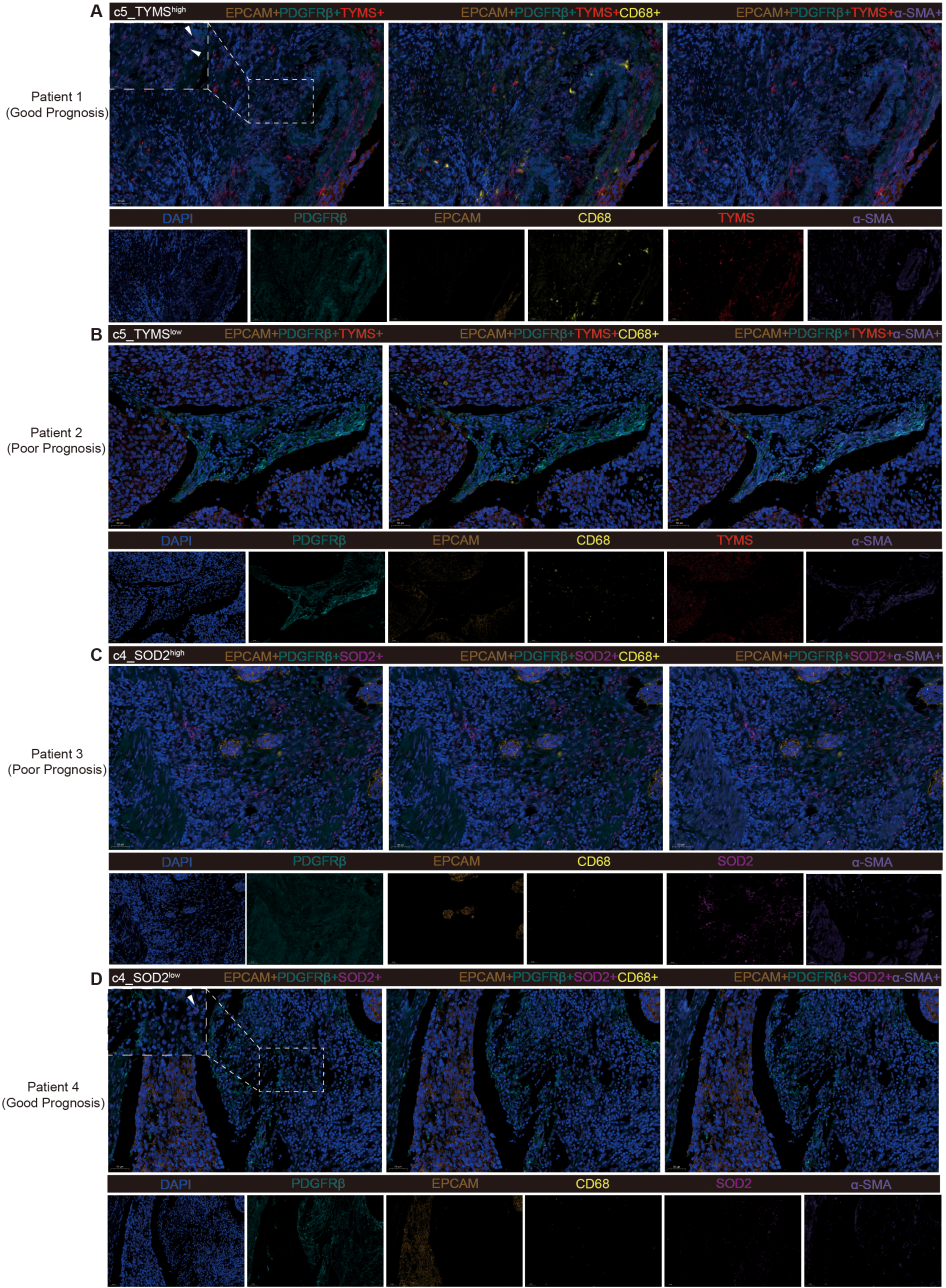
Multiple fluorescence staining of tissues samples from ESCC patients. **(A, B)** Validation of high and low expression level of c5_TYMS in ESCC tumor tissues samples and spatial distribution with microphages and fibroblasts. **(C, D)** Validation of high and low expression of level c4_SOD2 in ESCC tumor tissue samples and spatial distribution with microphages and fibroblasts.

To validate the accuracy of the analytical results in tumor tissues from ESCC patients, we selected for mIF staining, based on postoperative survival time, surgical specimens from 2 patients who died after recurrence within 1 year (poor prognosis) and 2 patients who had not yet produced a recurrence within 1 year (good prognosis), none of whom had received therapeutic treatment such as neoadjuvant therapy. First, both pericyte subpopulations of c5_TYMS and c4_SOD2 analyzed previously were validated in mIF. As mentioned previously, high c5_TYMS expression was associated with good prognosis, whereas high c4_SOD2 expression was associated with poor prognosis. Therefore, we compared them in tumor tissue samples from patients with different prognoses. c5_TYMS expression was higher in patients with good prognosis, and the opposite was true for those with poor prognosis (**Figures 5A, B**); c4_SOD2 expression was lower in patients with good prognosis, and the opposite was true for those with poor prognosis as well (**Figures 5C, D**), which was consistent with the results of the survival analysis Consistent. In addition, to observe the spatial distribution of these two pericyte subpopulations with immune cells, we stained macrophages and fibroblasts. It was found that there was more macrophage infiltration around c5_TYMS compared to c4_SOD2, while the difference in fibroblasts was minimal, with little or no difference in fibroblasts around c5_TYMS than around c4_SOD2 overall. This is in general agreement with our cellular communication results.

### 3.6 Molecular docking results of predicted drugs and proteins encoded by target genes

**Figure 6:**
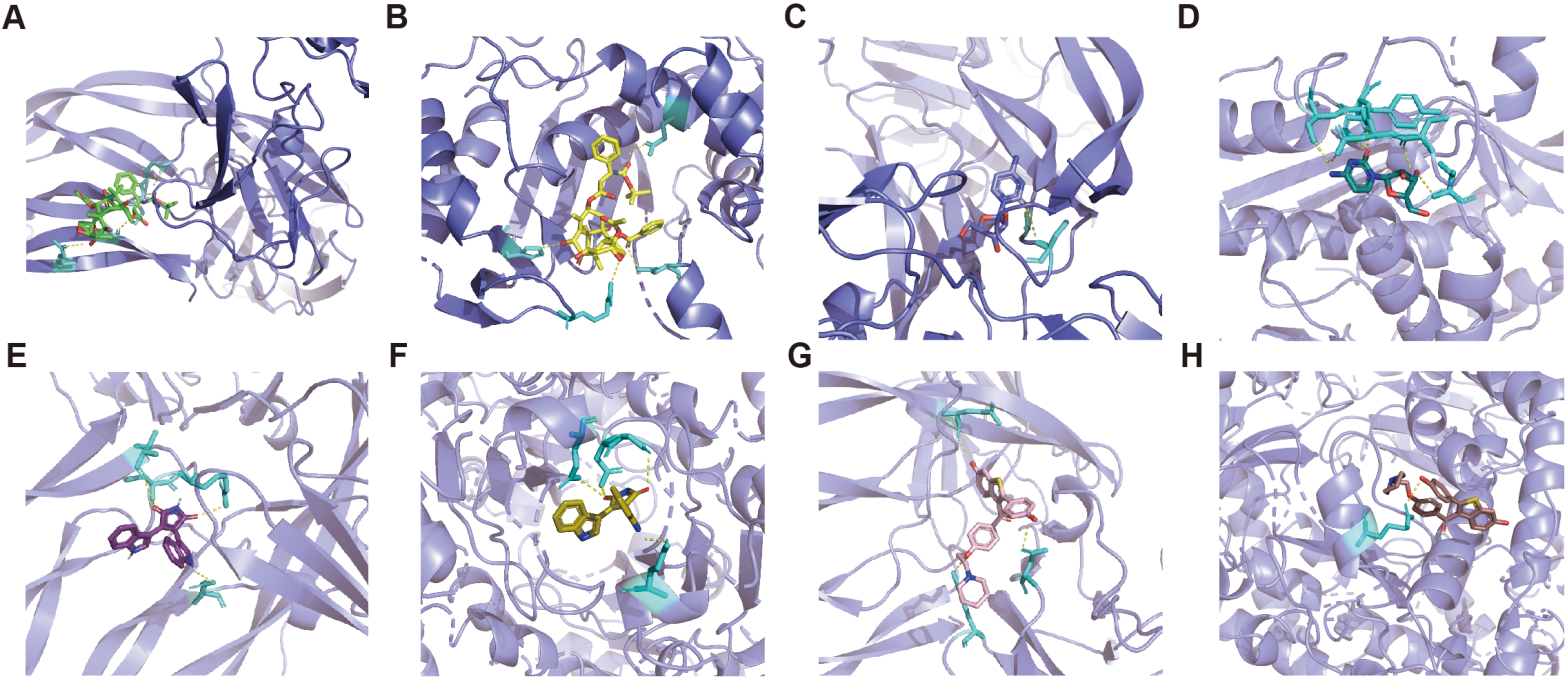
Molecular docking results of predicted drugs and protein encoded by target genes. **(A)** PDGFRβ and docetaxel. **(B)** TYMS and docetaxel. **(C)** PDGFRβ and cytarabine. **(D)** TYMS and cytarabine. **(E)** PDGFRβ and bisindolylmalemide. **(F)** SOD2 and bisindolylma-leimide. **(G)** PDGFRβ and raloxifene. **(H)** SOD2 and raloxifene.

The mIF results showed that TYMS and SOD2 were expressed to varying degrees in other constituent cells in the TME. We utilize the DSigDB database in the Enrichr database to predict small molecule drugs that may act on drug target genes. Therefore, in order to predict drugs that can specifically modulate pericyte subpopulations, only drugs with both PDGFRβ and TYMS or SOD2 targets are eligible. Drugs with both PDGFRβ and TYMS targets include docetaxel and cytarabine, while drugs with both PDGFRβ and SOD2 targets are bisindolylmaleimide and raloxifene (adjusted p-value less than 0.05). The PDB ID of the target protein, the PubChem ID of the drug, and the docking of the two of the target proteins, the PubChem IDs of the drugs, and the lowest molecular binding energies of the two docked are shown in **Table 1**. A lower molecular binding energy represents a stronger binding affinity of the small molecule drug to the protein. A score of less than -5.0 kcal/mol indicates potential binding and a score of less than -7.0 kcal/mol indicates a stronger binding affinity. Each medication candidate connects to its protein target via visible hydrogen bonds interactions. In addition, the binding pocket of each target was successfully occupied by four drug candidates. From the results, the drug with a better effect on c5_TYMS was docetaxel (binding energy = -8.1, -8.7 kcal/mol); raloxifene may be more effective against c4_SOD2, although raloxifene has a slightly lower binding energy to SOD2 (-6.4 kcal/mol), it has a higher binding energy to PDGFRβ (-8.1 kcal/mol), which implies that this drug has a better specificity for pericyte subpopulations and a weaker effect on the other constituent cells in the TME. The corresponding molecular docking diagram is displayed in **Figure 6**.

**Table 1:** Docking results of available proteins with small molecules.

Target	PDB ID	Drug	PubChem ID	Adjusted p-value	Binding energy
PDGFRβ	3MJG	Docetaxel	148124	0.014	-8.1
TYMS	1YPV	Docetaxel	148124	0.014	-8.7
PDGFRβ	3MJG	Cytarabine	6253	0.014	-6.0
TYMS	1YPV	Cytarabine	6253	0.014	-6.4
PDGFRβ	3MJG	Bisindolylmaleimide	2399	0.004	-7.1
SOD2	3LSU	Bisindolylmaleimide	2399	0.004	-7.0
PDGFRβ	3MJG	Raloxifene	5035	0.016	-8.1
SOD2	3LSU	Raloxifene	5035	0.016	-6.4

The lower the Binding Energy, the better the binding effect and the higher the affinity.

## 4 Discussion

In this study, we explored for the first time the pericyte subtypes in the ESCC tumor microenvironment based on large-sample single-cell sequencing data and revealed their heterogeneity, including the differences in the major functions exerted, the differences in the upstream and downstream regulatory networks, and the differences in the communication between different pericytes and other cellular subpopulations in the tumor microenvironment. This is of great significance for enriching the ESCC tumor microenvironment and thus targeting anti-tumor treatment strategies.

We first performed a functional enrichment analysis in all pericytes, which showed that pericytes were mainly enriched in pathways such as lymphocyte differentiation, regulation of activation, EMT, and regulation of immune system processes. Recently, several compelling studies have illustrated a bidirectional dialogue between TAMs and vascular cells, including pericytes (4, 16, 17). Pericytes can induce infiltration and polarization of M2 phenotype macrophages through direct or indirect effects, whereas TAMs can recruit pericytes in this process to orchestrate the formation of perivascular niches and promote tumor angiogenesis. This is consistent with the function we enriched for. In contrast, existing studies of pericytes and EMT point to the fact that EMT leads to the transformation of many markers in epithelial cells, including an increase in pericyte-like markers (N-cadherin, PDGFRβ and NG2). This confers pericyte-like properties, morphology and function to cancer cells. They can then aggregate in localized blood vessels and connect with vascular endothelial cells, thereby promoting vascular invasion and metastasis (18–20).

To better predict the functions that pericytes might play, we performed a re-clustering analysis and annotated six subpopulations based on their differential gene expression. By functional enrichment analysis, we found that these pericyte subpopulations do have subtle differences in function between them, i.e., each subpopulation crosses over in terms of the generalized function of pericytes, but each subpopulation predominantly performs a certain subclass of function. We first focus on the 4 subtypes that were not statistically significant when combined with the prognostic analysis. Based on the enrichment scores and the number of related pathways to be analyzed together, we found that c1_ARHGDIB mainly performs epithelial-mesenchymal transition (EMT)-related functions. c2_BCAM functions are mainly related to the regulation of cellular metal-ion transport, and the activity of ion transmembrane transporter, DNA-binding transcription factors. It has been found that inhibition of BCAM impairs KRAS-mutant colon cancer cells’ specific adhesion to endothelial cells, but not to pericytes, but the mechanism is not known (21). Coincidentally, c2_BCAM does have the weakest cellular communication with endothelial cells of the several isoforms, which may have some relevance to this study. c3_LUM primarily performs functions related to protein folding, disassembly, and binding. We did not find a predominantly dominant function in c6_KRT17, a subpopulation that functionally resembles more of a composite of the previous isoforms, and we speculate that this isoform may play a stabilizing and balancing role for pericytes as a whole. Based on the major functions of these four subtypes, we named them epithelial-mesenchymal transition cancer-associated pericytes, ionic cancer-associated pericytes, protein cancer-associated pericytes, and general cancer-associated pericytes.

We then look at those two subpopulations that have significant prognostic relevance. c4_SOD2 plays a major role in regulating immune cell development, activation, and adhesion, and it has been found that pericytes with high SOD2 expression enhance their anti-inflammatory effects (22, 23). SOD2 itself is also an inflammatory factor, but we mentioned earlier that c4_SOD2 is mainly enriched for TNF-γ responses in GSEA, is this a contradiction? Not really, because TNF-γ drives both immune activating and immunosuppressive effects, and interferon-gamma induces anti-tumor or pro-tumor mainly depending on the duration (acute or chronic) and magnitude of TNF-γ signaling, which is largely determined by the tumor load and immune cell infiltration status. Initially TNF-γ works by recruiting cohorts (CXCL9, CXCL10, CXCL11, and CXCR3) to promote antigen presentation (MHC class I and class II), T-cell initiation and activation (CD80, CD86, and CD40), and tumor cell killing (Fas and FASL). However, prolonged exposure to interferon-gamma transforms teammates into adversaries, producing pro-tumorigenic effects through immunosuppression (PDL1, IDO1, Fas, and FASL), angiogenesis (CXCL9, CXCL10, CXCL11, IDO1, and iNOS), and tumor cell proliferation (24–26). Combined with the prognostic analysis, we found that ESCC patients with high expression of c4_SOD2 had a poor prognosis, so we named them promoted cancer-associated pericytes (pro-CAPs), however, because c4_SOD2 may have dual anti-inflammatory and anti-tumor effects, we thought it would be more accurate to tentatively name it as double effect cancer-associated pericytes is more accurate. It has been demonstrated that the interaction between pericytes and endothelial cells affects tumor angiogenesis, and in combination with the strong communication between c4_SOD2 and endothelial cells, we hypothesize that c4_SOD2 is the key isoform of pericytes that promotes tumor vascular integrity (27–29). Whereas c5_TYMS mainly plays functions related to mitosis and cell cycle regulation, patients with high c5_TYMS expression have better survival, which we named as supportive cancer-associated pericytes (sup-CAPs), and based on the predicted functions as cell cycle cancer-associated pericytes based on their predictive function.

By analyzing the pericyte subpopulation profiles contained in all patients in the samples, we found that c1_ARHGDIB and c2_BCAM accounted for the major portion and were present in a certain proportion in both tumor tissue and normal tissue samples. This suggests to us that these two subgroups may not be significantly associated with ESCC tumor progression, metastasis, and recurrence, but as we know, tumors are heterogeneous, and the heterogeneity is even greater among different organs. So, it cannot be ruled out whether they play an important role in other organs or diseases. The proportion of the remaining 4 subtypes in the tissue samples is still somewhat different, some patients have all the pericyte subtypes in their tumor tissues, while some have only 1-4 of them, which is what we need to pay close attention to when we are looking for targets for our precision therapies, especially immunotherapy.

The above is only an analysis and prediction based on single-cell sequencing data, and the existing research on tumors and pericytes is very limited. There is an urgent need to validate the functions of these pericyte subtypes and their involvement and impact on prognosis through real-world patient tissue samples as well as basic experiments.

The present study predicts that docetaxel may have a modulatory effect on c5_TYMS. An analysis based on clinical trial data indicated that docetaxel not only maintained lymphocyte numbers and function, but also led to a significant increase in soluble immunomodulatory factor levels (30). It was also found that paclitaxel induced the release of cytotoxic extracellular vesicles from T-cells, thereby enhancing both T-cell receptor (TCR)-dependent and non-dependent cancer cell killing (31). This is consistent with our prediction that possibly promoting c5_TYMS expression is part of the picture. Raloxifene is currently used to treat breast cancer primarily through estrogen, and its role in affecting tumor immunity has rarely been reported. These drugs need to be explored in further experiments.

For a long time, pericytes have been neglected or not carefully studied because of their low percentage in TME. The results of the present study show that pericytes not only have multiple subtypes and different functions among them, but even the expression of individual subtypes is significantly associated with the prognosis of ESCC patients. Each patient’s own immune microenvironment is very different, which suggests that each component of the TME is worth exploring and researching, and each target or combination of targets should be explored, so as to maximize the potential of the existing drugs, develop new targeted drugs with stronger specificity, and achieve truly individualized treatment in the future.

## 5 Conclusions

The present study identified and discovered pericyte subpopulations that were significantly associated with prognosis, which provides new biomarkers for predicting patient prognosis and adds usable targets for immunotherapy, it is also important for gaining insights into the composition of the TME in ESCC.

## Data Availability

The original contributions presented in the study are included in the article/**Supplementary Material**. Further inquiries can be directed to the corresponding author.
