# Supplementary figures and images for: Single-cell sequencing analysis reveals cancer-associated pericyte subgroup in esophageal squamous cell carcinoma to predict prognosis
Source: Front Immunol. 2025 Jan 6;15:1474673. doi: 10.3389/fimmu.2024.1474673 (PMC11743493; doi:10.3389/fimmu.2024.1474673)

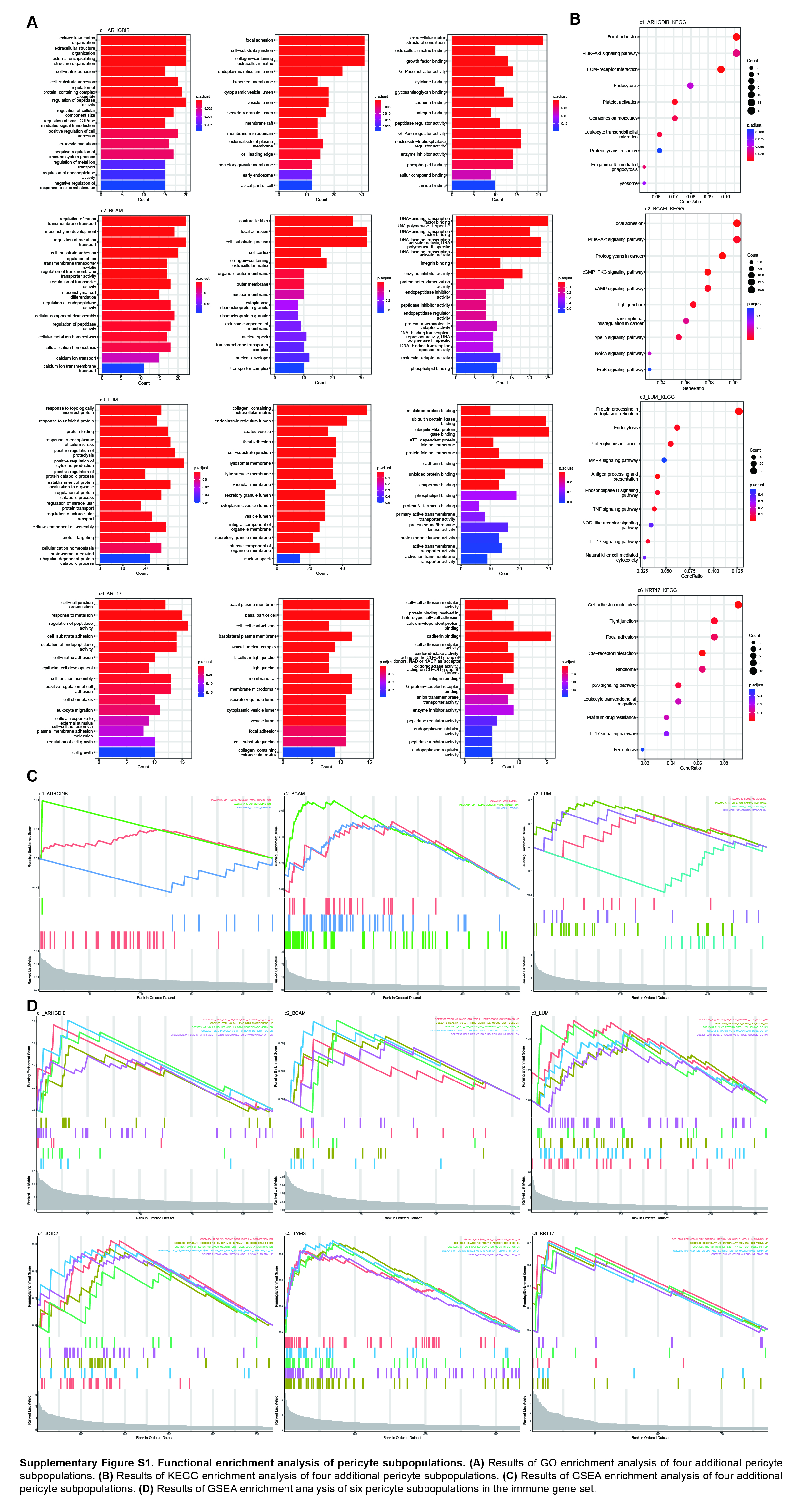

Supplement: Supplementary file 1 [file Image1.jpeg]

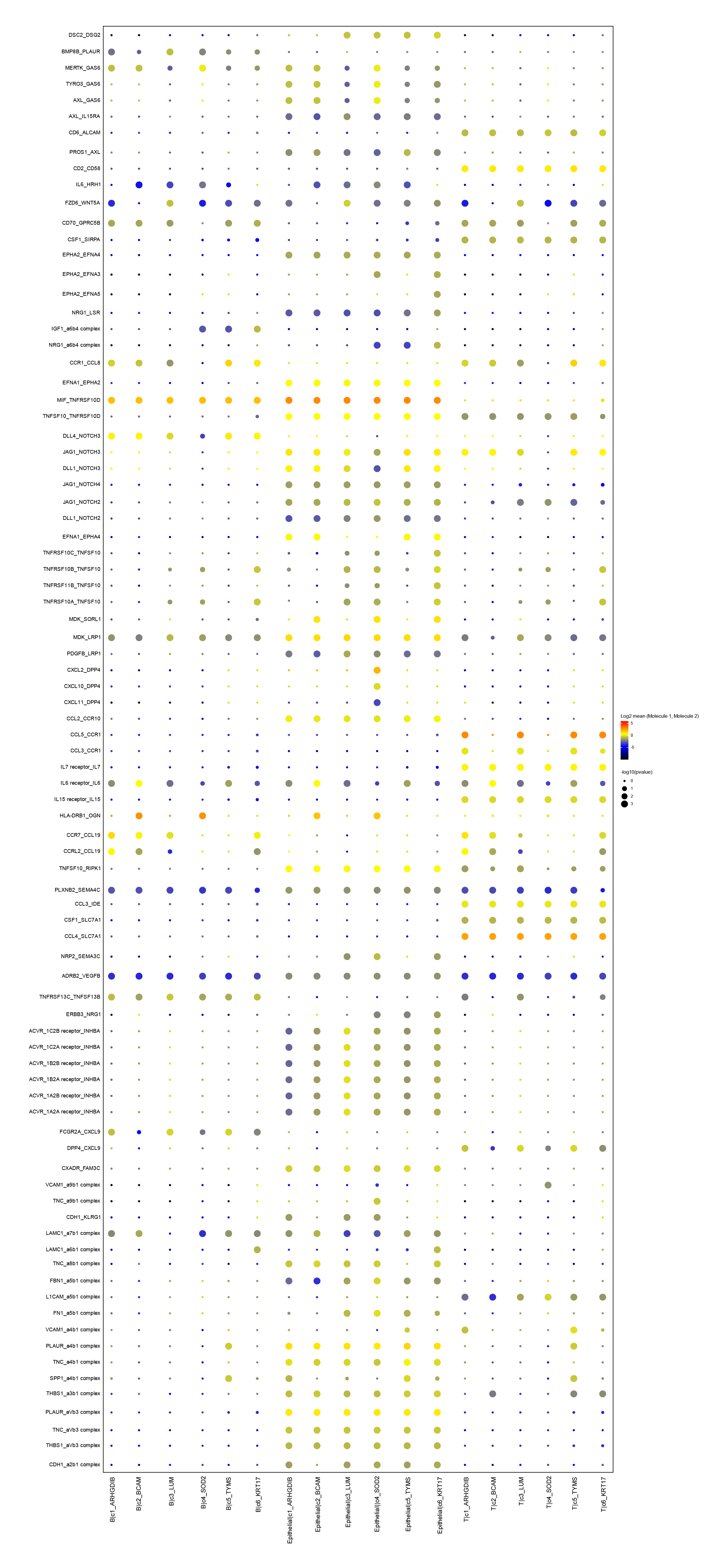

Supplement: Supplementary file 2 [file Image2.jpeg]

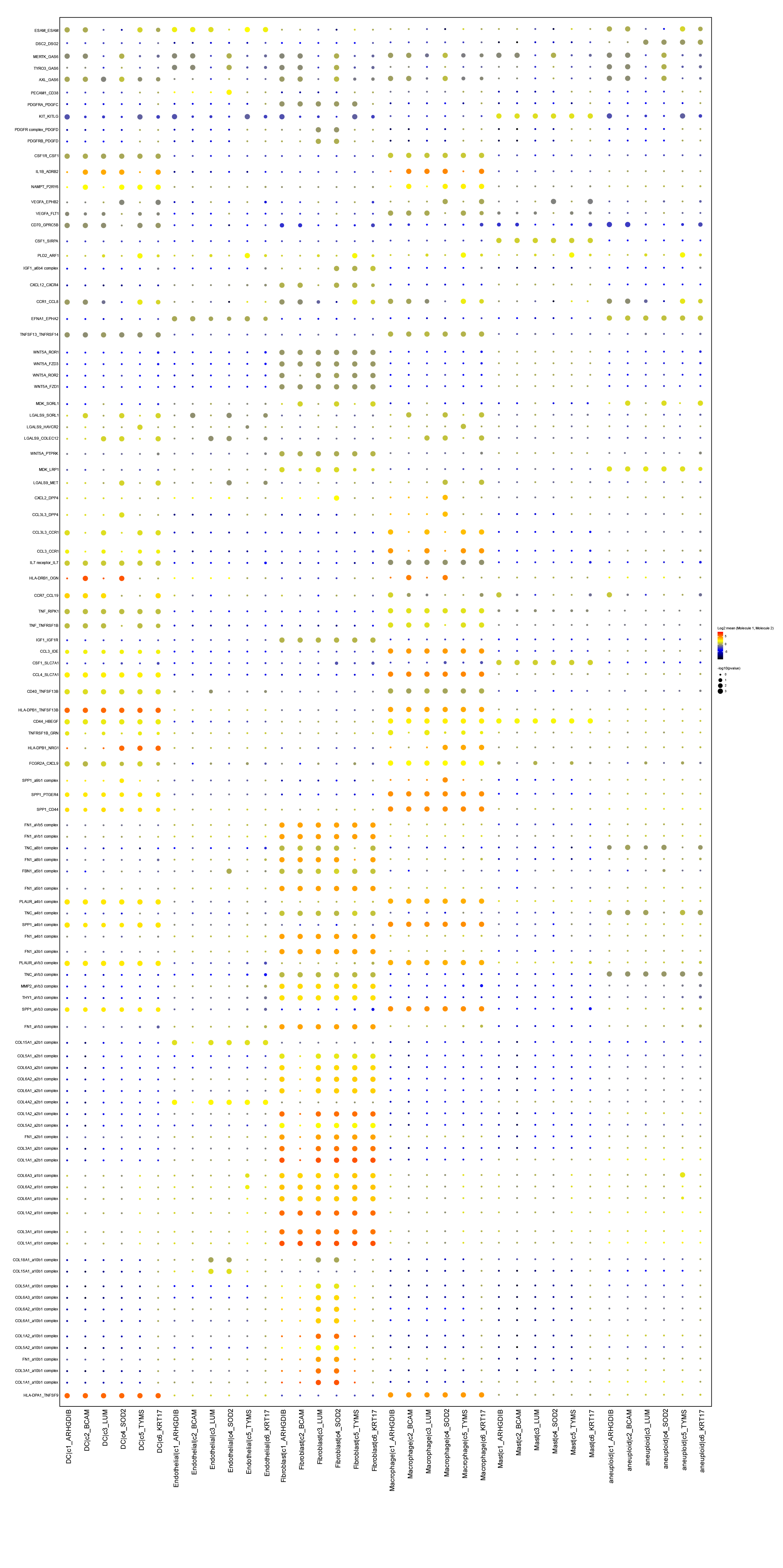

Supplement: Supplementary file 3 [file Image3.jpeg]
